# Molecular signatures identify immature mesenchymal progenitors in early mouse limb buds that respond differentially to morphogen signaling

**DOI:** 10.1242/dev.173328

**Published:** 2019-05-28

**Authors:** Robert Reinhardt, Fabiana Gullotta, Gretel Nusspaumer, Erkan Ünal, Robert Ivanek, Aimée Zuniga, Rolf Zeller

**Affiliations:** 1 Developmental Genetics, Department of Biomedicine, University of Basel, 4058 Basel, Switzerland; 2 Development and Evolution, Centro Andaluz de Biología del Desarrollo, Universidad Pablo de Olavide, 41013 Sevilla, Spain; 3 Swiss Institute of Bioinformatics, 4058 Basel, Switzerland; 4 Bioinformatics Core Facility, Department of Biomedicine, University of Basel, 4056 Basel, Switzerland

**Keywords:** Cell sorting, Chondrogenesis, Mesenchymal progenitors, Mouse limb bud, Signaling, Transcriptome

## Abstract

The key molecular interactions governing vertebrate limb bud development are a paradigm for studying the mechanisms controlling progenitor cell proliferation and specification during vertebrate organogenesis. However, little is known about the cellular heterogeneity of the mesenchymal progenitors in early limb buds that ultimately contribute to the chondrogenic condensations prefiguring the skeleton. We combined flow cytometric and transcriptome analyses to identify the molecular signatures of several distinct mesenchymal progenitor cell populations present in early mouse forelimb buds. In particular, jagged 1 (JAG1)-positive cells located in the posterior-distal mesenchyme were identified as the most immature limb bud mesenchymal progenitors (LMPs), which crucially depend on SHH and FGF signaling in culture. The analysis of gremlin 1 (*Grem1*)-deficient forelimb buds showed that JAG1-expressing LMPs are protected from apoptosis by GREM1-mediated BMP antagonism. At the same stage, the osteo-chondrogenic progenitors (OCPs) located in the core mesenchyme are already actively responding to BMP signaling. This analysis sheds light on the cellular heterogeneity of the early mouse limb bud mesenchyme and on the distinct response of LMPs and OCPs to morphogen signaling.

## Abstract (precis)

**Summary:** Cell sorting identifies limb bud mesenchymal progenitors (LMPs) with distinct molecular signatures and differential dependence on morphogen signaling. Specifically, two immature LMP populations with strong chondrogenic differentiation potential are identified.

## INTRODUCTION

The developing vertebrate limb bud is an excellent model for studying the molecular mechanisms and cellular interactions that govern proliferative expansion, specification and differentiation of mesenchymal progenitors during organogenesis. The limb bud mesenchymal progenitors (LMPs) will give rise to the osteo-chondrogenic lineages of the appendicular skeleton, tendons and connective tissue. In contrast, muscles arise from myoblasts that migrate from the somites into the early limb bud (reviewed by Zuniga, 2015). There is evidence that the mesenchyme consists of molecularly distinct anterior and posterior compartments during the initiation of limb bud development (Osterwalder et al., 2014). It has been shown that SHH morphogen signaling specifies the antero-posterior (AP) identities of the future digits during the onset of mouse limb bud outgrowth (around embryonic day E9.75-E10.5; Zhu et al., 2008). In parallel, a feedback signaling system is established between the posterior SHH signaling center and FGF signaling by the apical ectodermal ridge (AER), which regulates the survival and proliferative expansion of LMPs in concert with gremlin 1 (GREM1)-mediated BMP antagonism and WNT signaling (ten Berge et al., 2008; Zhu et al., 2008; Benazet et al., 2009). In contrast, much less is known about the cellular heterogeneity of the mesenchyme and potential differences in the mesenchymal response to morphogen signaling.

LMPs arise by a local epithelial-to-mesenchymal transition (EMT) of the coelomic epithelium within the presumptive limb field, which is regulated by the TBX5 transcriptional regulator and FGF10 signaling (Gros and Tabin, 2014). Experimental analysis and model simulations show that distal progression of limb bud outgrowth is driven by oriented, rather than random, cell behaviors and division (Boehm et al., 2010; Gros et al., 2010). Lineage tracing identified a dorso-ventral compartment boundary that overlaps with the dorso-ventrally restricted expression of specific genes in mouse limb buds (Arques et al., 2007). Furthermore, genetic mapping of the descendants of *Shh*-expressing cells showed that they give rise to the two posterior-most and part of the central digit (Harfe et al., 2004). The *Shh*-expressing mesenchymal cells were isolated from mouse limb buds by means of an EGFP marker in combination with fluorescence-activated cell sorting (FACS). Their analysis identified the cistrome and gene expression signature of the SHH-signaling cells in the posterior limb bud mesenchyme (Rock et al., 2007; VanderMeer et al., 2014). As limb bud outgrowth progresses, the distal and sub-ectodermal mesenchyme is kept in a proliferative and undifferentiated state by AER-FGF and ectodermal WNT signaling (Pearse et al., 2007; ten Berge et al., 2008; Karamboulas et al., 2010). In contrast, the core mesenchyme expresses the SOX9 transcriptional regulator, which marks the osteo-chondrogenic progenitors (OCPs) from early stages onwards (Akiyama et al., 2005). In particular, SOX9 controls the mesenchyme to chondrocyte transition and initiation of chondrogenic differentiation (Wright et al., 1995; Barna and Niswander, 2007). FACS analysis of *Sox9*-EGFP-positive and -negative cells from mouse handplates (E11.5) showed that digit progenitors express SOX9 in a periodic pattern (Raspopovic et al., 2014).

Here, we first investigated the cell cycle kinetics, which showed that the percentage of mesenchymal cells in S phase decreases in parallel to the increase in cell numbers during progression of forelimb bud outgrowth. By combining flow cytometry with RNA sequencing (RNA-seq) analysis, we were able to identify and analyze distinct cell mesenchymal cell populations in early mouse forelimb buds. This analysis identified distinct immature LMPs located in the posterior-distal and peripheral mesenchyme and osteo-chondrogenic progenitors (OCPs) in the core mesenchyme. One of the three LMP populations encompasses myoblasts, while the other two represent distinct LMP populations with chondrogenic differentiation potential in culture. Comparative functional analysis showed that the transcriptional response to morphogenetic signaling differs significantly among the two chondrogenic LMP populations and OCPs. Genetic analysis of early forelimb buds revealed that the survival of the most immature LMPs located in the posterior-distal mesenchyme depends crucially on GREM1-mediated BMP antagonism.

## RESULTS

### Quantitation of limb bud mesenchymal cell number and cell cycle kinetics during forelimb bud outgrowth

**Fig. 1.**
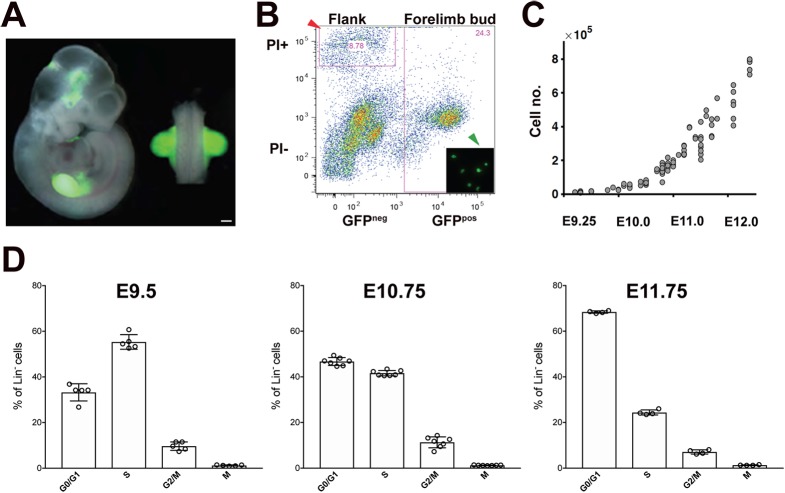
**Forelimb bud mesenchymal cell numbers and cell cycle analysis.** (A) *Prx1-*Cre was used to activate EGFP under control of the β-actin locus (βactGFP) in the forelimb bud mesenchyme. A representative embryo at E10.75 (37 somites) is shown. Scale bar: 250 μm. (B) Representative FACS analysis of *Prx1-*Cre/βactGFP forelimb buds. Necrotic and apoptotic cells are electronically gated in the upper part (red arrowhead), while the EGFP-positive cells are gated in the right half. Fluorescence microscopy confirmed that single EGFP-positive cells were analyzed (green arrowhead). Results shown are representative of *n*≥3 samples. (C) Experimentally determined forelimb bud mesenchymal cell numbers from accurately somite staged embryos between E9.5 and E12.0. Individual data points are shown. See also Table 1. (D) Analysis of the cell cycle and mitotic cells in lineage-negative (Lin^−^) mesenchymal cells from mouse forelimb buds at E9.5 (24-28 somites, *n*=5), E10.75 (35-39 somites, *n*=7) and E11.75 (49-52 somites, *n*=4). Data are mean±s.d.

Mouse forelimb bud mesenchymal cell numbers were determined by *Prx1-*Cre-mediated activation of an EGFP transgene in the endogenous β-actin locus. This results in EGFP expression by the vast majority of all limb bud mesenchymal cells (Fig. 1A; Logan et al., 2002; Jägle et al., 2007). Single cells were prepared from dissected forelimb buds (inset, Fig. 1B), which resulted in 8-15% cell death at all stages (red arrowhead, Fig. 1B). Forelimb buds were accurately staged by counting somite numbers, and mesenchymal cell numbers were determined by counting the EGFP-positive cells in defined sub-fraction volumes using flow cytometry (for details, see Materials and Methods, Fig. 1C, Table 1). Mesenchymal cell numbers increased from ∼1×10^4^ (22 somites at E9.25) to ∼7.7×10^5^ cells (54 somites at E12.0, Fig. 1C and Table 1). Cell numbers during the onset of forelimb bud development (18 somites at E9.0) were determined by counting DAPI-stained mesenchymal cell nuclei on serial sections using stacks of confocal images. This analysis established that forelimb buds at E9.0 contain ∼4512±974 mesenchymal cells (mean±s.d.; *n*=4).

**Table 1.**
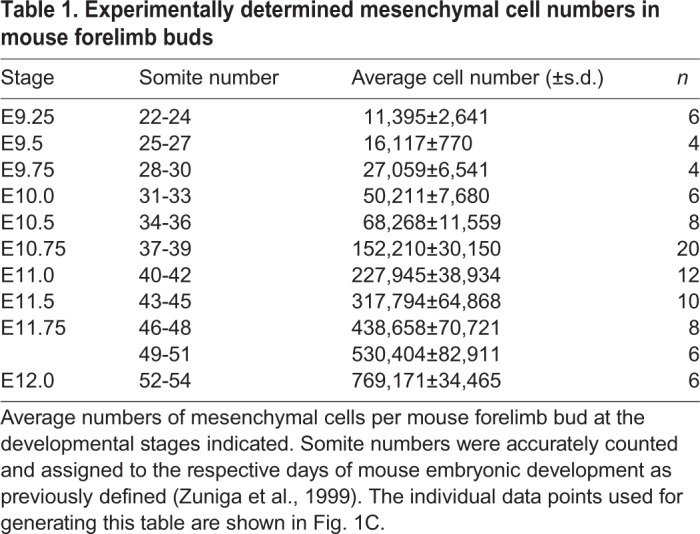
**Experimentally determined mesenchymal cell numbers in mouse forelimb buds**

The cell cycle kinetics at the three stages of forelimb bud outgrowth were analyzed using flow cytometric determination of cellular DNA contents and mitotic cells by measuring propidium iodide uptake and detecting phospho-histone H3-positive cells, respectively (Fig. 1D). This analysis revealed that the fraction of mesenchymal cells in S phase was highest at E9.5, whereas the fraction of mesenchymal cells in the G0/G1-phase of the cell cycle was highest at E11.75. In contrast, the fraction of mitotic cells was similar at all stages (mean at E9.5, 1.2%; E10.75, 1.22%; E11.75, 1.31%, Fig. 1D). This decrease in the fraction of cells in S phase was independently confirmed by directly assessing DNA synthesis by BrdU incorporation, which was highest in the mesenchyme of early limb buds (Fig. S1). Taken together, this analysis showed that mitotic rates are similar at all stages, but in early forelimb buds more than half of all mesenchymal cells are in S phase (E9.75), while at late stages most are in the G0/G1 phase of the cell cycle (∼68%, E11.75, Fig. 1D).

### FACS identifies distinct mesenchymal progenitor populations in early mouse forelimb buds (E10.5-E10.75)

**Fig. 2.**
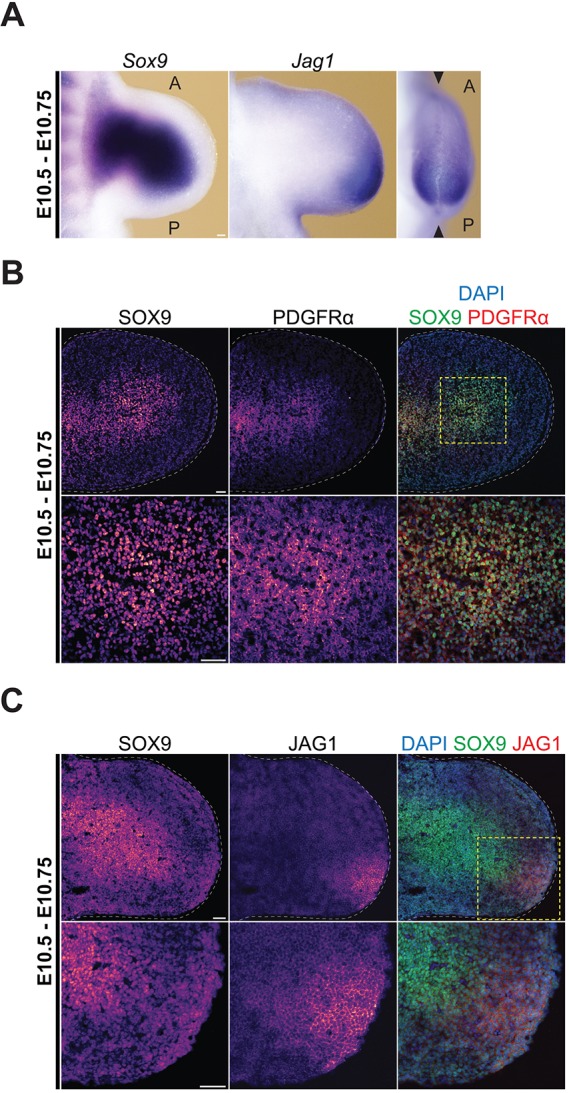
**Key markers used to identify mesenchymal cell populations in mouse forelimb buds.** (A) Whole-mount RNA *in situ* hybridization shows the spatial distribution of the *Sox9* transcription factor and the Notch ligand jagged 1 (*Jag1*) in forelimb buds at E10.5-E10.75 (35-38 somites). (B,C) Immunohistochemistry using forelimb buds at E10.5-10.75 detects the platelet-derived growth factor receptor α (PDGFRα), SOX9 and JAG1 proteins in sagittal sections through the limb bud apex (the approximate plane of section is indicated by arrowheads in the right-most panel in A). The right panels show the colocalization of PDGFRα and SOX9 in the core mesenchyme (B), while JAG1-positive cells are located in the posterior-distal SOX9-negative mesenchyme (C). Images are pseudo-colored in accordance with fluorescence intensity (purple, low; yellow, high). White dashed lines outline limb buds. Yellow dashed lines indicate the regions shown in more detail below. A, anterior; P, posterior. Scale bars: 50 µm.

**Fig. 3.**
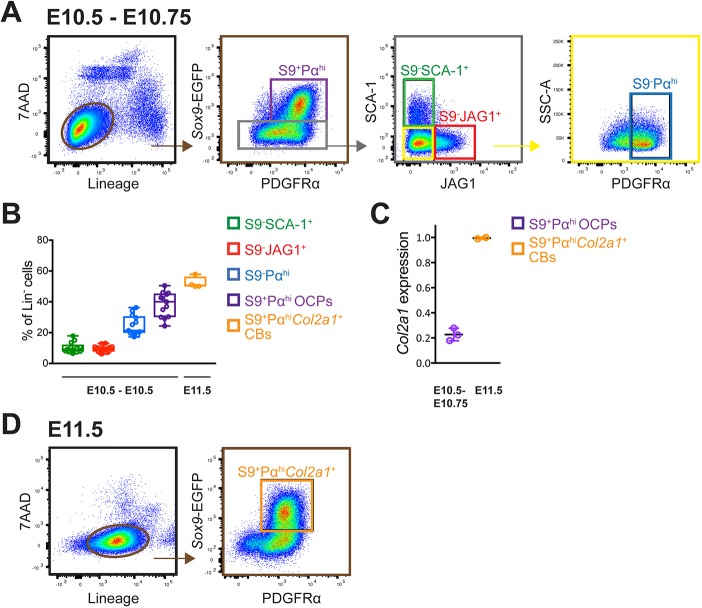
**Isolation of distinct mesenchymal cell populations from forelimb buds at E10.5-E10.75 and E11.5.** (A) FACS strategy to isolate the different cell populations from lineage-negative (Lin^−^) mesenchymal cells of mouse forelimb buds (35-38 somites, E10.5-E10.75, first panel). S9^+^Pα^hi^ (violet): *Sox9*-EGFP-positive cells corresponding predominantly to osteo-chondrogenic progenitors (OCPs) express high levels of PDGFRα. In addition, three mesenchymal populations of *Sox9*-EGFP-negative cells were isolated: S9^−^SCA-1^+^ (green), S9^−^JAG1^+^ (red) and S9^−^Pα^hi^ (blue) cells. (B) Box and whisker plot showing the abundance (%) of the different cell populations within the Lin^−^ mesenchymal cells. The midline represents the median, the upper and lower limits of the box indicate the first and third quartiles, and the whiskers indicate the lowest and highest values, respectively. (C) Relative *Col2a1* expression levels in S9^+^Pα^hi^ OCPs (E10.5-E10.75) and S9^+^Pα^hi^*Col2a1*^+^ chondroblasts (CBs, E11.5). (D) CB were isolated from Lin^−^ forelimb bud mesenchymal cells at E11.5 as S9^+^Pα^hi^ cells that express *Col2a1*: S9^+^Pα^hi^*Col2a1*^+^ CBs (orange, see C for *Col2a1* expression). Representative FACS experiments are shown and the same gates were used for all analyses.

**Fig. 4.**
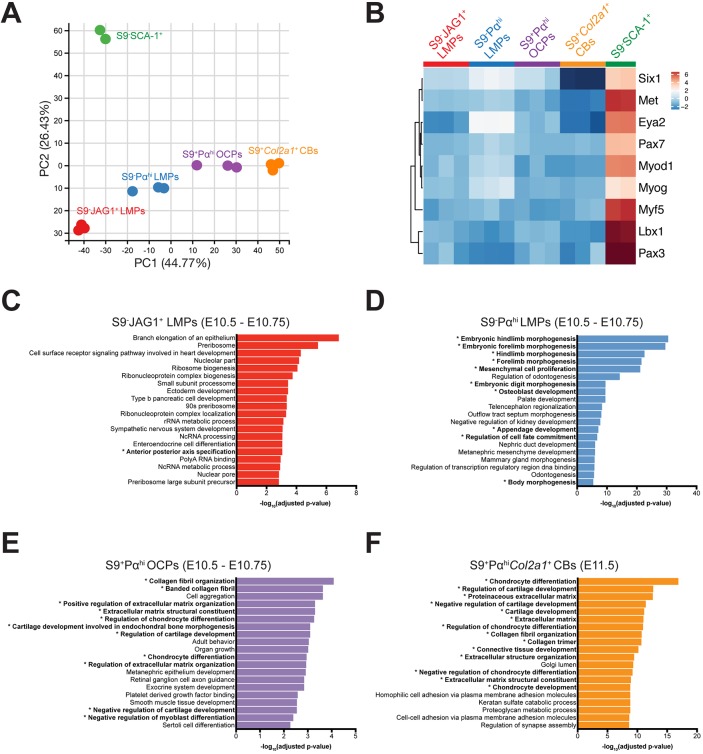
**Comparative transcriptome analysis identifies two early LMP populations.** (A) Principal components analysis (PCA) of RNA-seq metadata from the five different forelimb bud mesenchymal cell populations identified. Three biological replicates were sequenced for all populations, with the exception of S9^−^SCA-1^+^ cells, which yielded only two samples of sufficient sequencing quality. (B) Heatmap showing the relative expression levels of key genes involved in myoblast migration and differentiation. Analysis shows that the S9^−^SCA-1^+^ progenitors express the highest levels of myoblast-specific markers in comparison with the other populations (Tables S1 and S2). Higher than average expression, orange-red; lower than average, blue; average, white. (C-F) Global gene ontology (GO) enrichment analysis of the genes whose expression is higher in the cell population of interest than all other populations (Tables S2-S6). (C) S9^−^JAG1^+^ LMPs, (D) S9^−^Pα^hi^ LMPs, (E) S9^+^Pα^hi^ OCPs (all E10.5-E10.75) and (F) S9^+^Pα^hi^*Col2a1*^+^ chondroblasts (E11.5). The *x*-axis shows the -log_10_ of the false discovery rate (FDR). Asterisks indicate chondrogenesis- and limb-related GO terms.

**Fig. 5.**
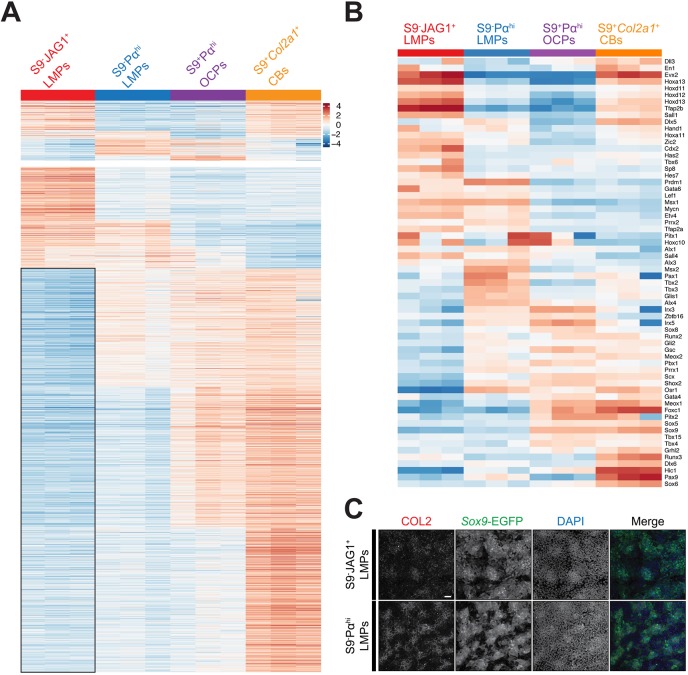
**S9^−^JAG1^+^ and S9^−^Pα^hi^ LMPs are early mesenchymal progenitors with distinct molecular signatures and robust chondrogenic differentiation potential.** (A) Comparative analysis of the transcriptomes based on pseudo-temporal ordering from S9^−^JAG1^+^ to S9^−^Pα^hi^ LMPs to S9^+^Pα^hi^ OCPs (E10.5-E10.75) and to S9^+^Pα^hi^*Col2a1*^+^ CBs (E11.5, Table S7). (B) Manually curated list of differentially expressed transcriptional regulators required for limb bud and/or limb skeletal development (Table S10) using the ‘Mammalian Phenotype Ontology Annotations’ related to limb development from Mouse Genome Informatics (www.informatics.jax.org/). Higher than average expression, orange-red; lower than average, blue; average, white. (C) Culturing FACS-isolated S9^−^JAG1^+^ and S9^−^Pα^hi^ LMPs for 24 h results in activation of *Sox9*-EGFP expression in cells that undergo aggregation to form the typical chondrogenic condensations. Scale bar: 50 µm.

**Fig. 6.**
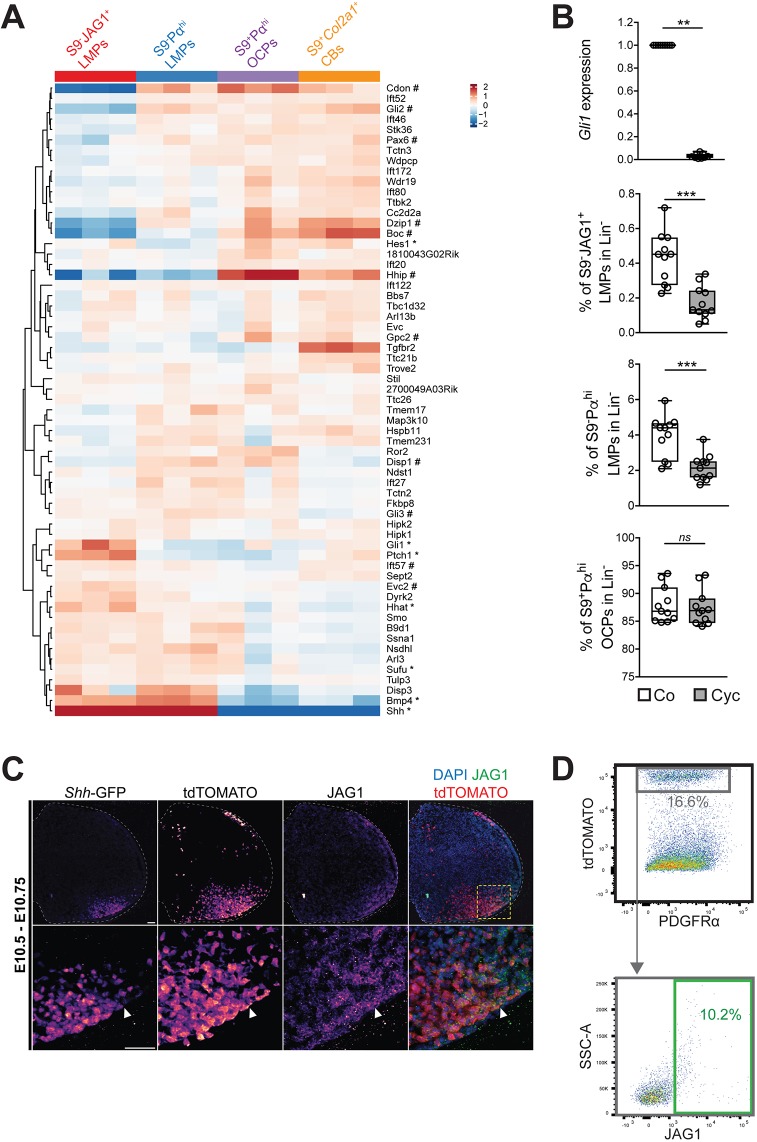
**S9^−^JAG1^+^ and S9^−^Pα^hi^ LMPs depend on SHH pathway activity.** (A) Heatmap showing expression level of genes associated with the term ‘Smoothened (SMO) signaling pathway’ (GO:0007224, Table S8). Known distally (*) and centrally (#) expressed genes are highlighted. (B) Limb bud mesenchymal cells (E10.5-E10.75) were cultured for 12 h either in normal medium (control, co) or in medium supplemented with 20 µM cyclopamine (Cyc, a SMO antagonist). The top panel shows the relative expression of *Gli1*, a direct transcriptional target of SHH signal transduction, as determined by RT-qPCR analysis. Lower three panels show FACS analysis illustrating that the fraction (%) of S9^−^JAG1^+^ and S9^−^Pα^hi^ LMPs was significantly reduced when SMO-mediated signal transduction was blocked. In contrast, the fraction of S9^+^Pα^hi^ OCPS was not altered. S9^−^JAG1^+^ LMPs were decreased from 0.43%±0.14% to 0.17±0.09% and S9^−^Pα^hi^ LMPs were decreased from 3.94±1.18% to 2.14±0.73% of the lineage-negative cells in culture. The midline represents the median, the upper and lower limits of the box indicate the first and third quartiles, and the whiskers indicate the lowest and highest values, respectively. The Wilcoxon test was used for statistical analysis of results: **P≤0.01, ***P≤0.001. (C) Distribution of *Shh*-expressing cells (*Shh*-GFP, white arrowheads indicate the distal border) and *Shh* descendants expressing tdTOMATO in a representative forelimb bud (E10.5-E10.75). This pattern arose from permanent activation of the *Rosa26*^tdTomato^ transgene by *Shh*^GFPCre^-induced recombination. The JAG1 protein was detected using specific antibodies. The overlap (right panel) shows that only a small fraction of cells co-expressed tdTOMATO (red) and JAG1 (green; *n*=3 independent samples). Images are pseudo-colored in accordance with fluorescence intensity (purple, low; yellow, high). White dashed lines outline the limb buds. Yellow dashed lines demarcate the regions shown in more detail. Scale bars: 50 µm. (D) FACS analysis confirmed that only a small fraction of tdTOMATO-positive cells co-expressed JAG1 (*n*=3).

**Fig. 7.**
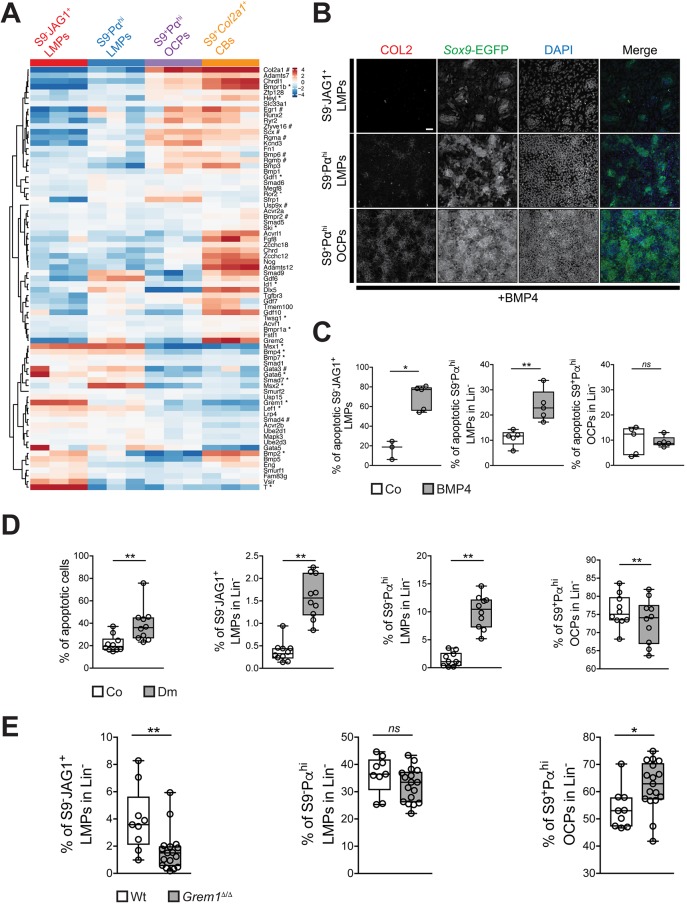
**Immature S9^−^JAG1^+^ LMPs depend crucially on GREM1-mediated BMP antagonism.** (A) Heatmap showing expression level of genes associated with the GO term ‘Cellular response to BMP stimulus’ (GO:0071773, Table S9). Known distal (*) and central (#) expressed genes are highlighted. (B) S9^−^JAG1^+^ and S9^−^Pα^hi^ LMPs and S9^+^Pα^hi^ OCPs were cultured for 24 h in medium supplemented with 10 ng/ml BMP4. Controls were cultured in medium with solvent. In all cases, equal numbers of live mesenchymal cells were plated after FACS isolation. Only S9^+^Pα^hi^ OCPs underwent robust chondrogenic differentiation within 24 h in BMP4-supplemented medium. Scale bar: 50 µm. (C) Quantitation of apoptotic cells in the three mesenchymal cell populations after culturing them for 24 h in BMP4-supplemented medium. While apoptosis was not altered for the OCP population, cell death was significantly increased for both LMP populations. (*n*≥3 per condition and cell type). (D) Forelimb bud mesenchymal cells (E10.5-E10.75) were cultured in medium supplemented with 5 µM dorsomorphin (Dm) to inhibit BMP signaling (Fig. S6A). Controls (Co) were cultured in medium supplemented with solvent only. After 12 h, the overall cellular apoptosis and the fractions (%) of S9^−^JAG1^+^ and S9^−^Pα^hi^ LMPs, and S9^+^Pα^hi^ OCPs were determined. Both LMP populations increased significantly, while the OCP population was reduced (*n*=10). (E) Comparative FACS analysis of the fraction (%) of S9^−^JAG1^+^ and S9^−^Pα^hi^ LMPs, and S9^+^Pα^hi^ OCPs in pairs of forelimb buds from wild-type (Wt) and *Grem1*-deficient mouse embryos (Grem1^Δ/Δ^) at E10.5-E10.75 (*n*=9 for *Grem1*^Δ/Δ^; *n*=17 for wild-type forelimb buds). The fraction of S9^−^JAG1^+^ LMPs was halved, while the fraction of S9^−^Pα^hi^ LMPs was not altered and the fraction of S9^+^Pα^hi^ OCPs increased in *Grem1*-deficient forelimb buds. The midline represents the median, the upper and lower limits of the box induacte the first and third quartiles, and the whiskers indicate the lowest and highest values, respectively, in the box and whisker plots shown in C-E. The Mann-Whitney test was used for statistical analysis of all results shown in C-E. *P≤0.05, **P≤0.01; ns, not significant.

Previous analysis of limb bud mesenchymal cells had established that the vast majority express platelet-derived growth factor receptor α (PDGFRα; Takakura et al., 1997), which, in combination with SCA-1, allowed FACS-mediated isolation of mesenchymal stromal cells (MSCs) during limb long bone development (Morikawa et al., 2009; Craft et al., 2013; Nusspaumer et al., 2017). To gain insight into the potentially cellular heterogeneity during the early phase of forelimb bud outgrowth, we used mouse embryos at E10.5-E10.75 (35-38 somites, Figs 2–7). These are the earliest forelimb bud stages that permitted isolation of sufficient mesenchymal cells for functional analysis. We used several cell surface markers suited for FACS analysis (Fig. 2) in combination with a *Sox9*-EGFP allele (*Sox9^IRES-EGFP^*; Chan et al., 2011), as SOX9 is expressed by the OCPs located in the core limb bud mesenchyme already at early stages (Fig. 2A-C). Immunofluorescence analysis also detected high levels of PDGFRα in the core mesenchyme, whereas levels were lower in the peripheral mesenchyme (Fig. 2B). In contrast, the SCA-1 transcript distribution in early forelimb buds has previously been shown to be rather diffuse with higher levels in the peripheral mesenchyme (Nusspaumer et al., 2017). Our analysis of different cell surface markers suited for FACS isolation also identified the transmembrane NOTCH ligand JAG1 due to its localized expression in the posterior-distal forelimb bud mesenchyme (E10.5-E10.75, Fig. 2A,C; Fig. S2; Panman et al., 2006). Based on these results, we decided to use these markers for FACS-mediated isolation of distinct forelimb bud mesenchymal cell populations (E10.5-E10.75, Fig. 3A). Initially apoptotic, ectodermal and various non-mesenchymal cell types were excluded from further analysis using the appropriate cell surface markers (exclusion of lineage-positive cells, for details see Materials and Methods). The vast majority of these lineage-negative (Lin^−^) cells were PDGFRα-positive (Pα^+^) mesenchymal cells, which were separated further into SOX9-positive (S9^+^) and -negative (S9^−^) cells by their *Sox9*-EGFP expression (second panel in Fig. 3A). The core mesenchymal cells expressing SOX9 and high levels of PDGFRα (S9^+^Pα^hi^, Fig. 2A-C) corresponded to ∼38±8% of all Lin^−^ mesenchymal cells (Fig. 3B). This population consisted predominantly of OCPs, as it expressed only low levels of *Col2a1*, a molecular indicator of chondroblast differentiation (Fig. 3C; Akiyama et al., 2005). The SOX9-negative Pα^+^cells (S9^−^Pα^+^ cells) were sorted further with respect to their expression of the SCA-1 and JAG1 antigens, leading to isolation of three additional populations (third panel in Fig. 3A). FACS analysis established these as distinct populations because there was no overlap between the SCA-1 (S9^−^SCA-1^+^) and JAG1 (S9^−^JAG1^+^) populations. The third population of SOX9-negative cells expressed neither SCA-1 nor JAG1, but high levels of PDGFRα (S9^−^Pα^hi^, right panel in Fig. 3A). This S9^−^Pα^hi^ population included ∼25±6% of all Lin^−^ mesenchymal cells, while the S9^−^SCA-1^+^ (∼6±2%) and S9^−^JAG1^+^ (∼9±21%) populations were much less abundant.

### Molecular signatures identify S9^−^Pα^hi^ and S9^−^JAG1^+^ LMPs as early progenitors with robust chondrogenic differentiation potential in culture

To gain insight into the transcriptional signatures of the three mesenchymal cell populations and OCPs isolated from mouse forelimb buds at E10.5-E10.75 (Fig. 3A), FACS was combined with RNA-seq analysis (Figs 4 and 5). Chondroblasts expressing high levels of *Col2a1* were isolated from forelimb buds at E11.5 (45-47 somites) as S9^+^Pα^hi^*Col2a1*^+^ cells (Fig. 3B-D) and included in the analysis to discriminate OCPs from chondroblasts. Principal components analysis (PCA) of the RNA-seq data showed that the biological replicates cluster well, pointing to minimal intra-group variability. This analysis revealed that the S9^−^SCA-1^+^ mesenchymal cell population exhibit the highest variance along the *y*-axis, indicating that these cells might be rather different from the other populations (Fig. 4A). As expected, OCPs (S9^+^Pα^hi^ population) were rather similar to chondroblasts (S9^+^Pα^hi^*Col2a1*^+^ population), whereas S9^−^JAG1^+^ and S9^−^Pα^hi^ LMPs differed from both OCPs and chondroblasts, but to a lesser extent than S9^−^SCA-1^+^ cells (Fig. 4A).

Next, the RNA-seq datasets were subjected to Gene Ontology (GO) analysis to identify the genes and pathways whose expression differed significantly in one population versus all others. GO analysis of the S9^−^SCA-1^+^ population revealed that genes and pathways functioning in cell growth/proliferation and metabolic processes were expressed at higher than average levels, while the expression of genes functioning during limb bud development and chondrogenesis were expressed at lower than average levels (Fig. S3A,B). In addition to S9^−^SCA-1^+^ cells being actively proliferating progenitors, the GO analysis revealed that they expressed genes functioning in migration and differentiation of myoblasts (Fig. 4B; Chal and Pourquie, 2017). These included the c-MET receptor tyrosine kinase (*Met*), myogenin (*Myog*), myogenic differentiation factor 1 (*Myod1*), myogenic factor 5 (*Myf5*), and the *Pax3* and *Pax7* transcriptional regulators (Fig. 4B). Furthermore, culturing S9^−^SCA-1^+^ cells under conditions that favor chondrogenesis resulted in their elimination by cell death rather than induction of chondrogenic differentiation (data not shown). Our gene expression data suggest that the S9^−^SCA-1^+^ cell population isolated from early forelimb buds (E10.5-E10.75) encompasses myogenic rather than chondrogenic progenitors.

S9^−^JAG1^+^ LMPs displayed much less variance along the *y*-axis and appeared more closely related to the other three populations than S9^−^SCA-1^+^ cells (Fig. 4A). GO analysis showed that genes functioning in pathways relevant to cell growth/proliferation, metabolism and diverse developmental processes were also expressed at higher than average levels (Fig. 4C). In addition, genes belonging to pathways functioning in limb bud, chondrogenic and skeletal development were expressed at low levels by S9^−^JAG1^+^ LMPs (Fig. S3C). Overall the GO analysis indicated that these cells either belong to a non-chondrogenic lineage or have not yet been determined, which would point to the immature state of S9^−^JAG1^+^ LMPs (see below). In contrast, S9^−^Pα^hi^ LMPs most prominently expressed genes with essential functions during limb bud morphogenesis and mesenchymal cell proliferation (Fig. 4D, Fig. S2D). As expected, S9^+^Pα^hi^ OCPs and S9^+^Pα^hi^*Col2a1*^+^ chondroblasts expressed high levels of genes functioning in chondrocyte and/or cartilage differentiation, extra-cellular matrix and collagen fibril organization. Conversely, the expression of genes functioning in cell growth/proliferation and metabolic processes was lower than average (Fig. 4E,F, Fig. S3E,F).

The global molecular differences among two LMP populations, OCPs and chondroblasts, were exemplified by the ordered comparison of all differentially expressed genes shown in Fig. 5A. The most striking feature of this comparison is the large cluster of genes expressed at lower than average levels in S9^−^JAG1^+^ LMPs (indicated by the black square in Fig. 5A). Although the expression of a significant fraction of these genes was increased in S9^−^Pα^hi^ LMPs, this was enhanced in S9^+^Pα^hi^ OCPs and S9^+^Pα^hi^*Col2a1*^+^ chondroblasts, with the latter expressing the vast majority at high levels (Fig. 5A). To gain further insight into the relatedness and functional relevance of these changes in the four cell populations, we manually curated a list of the differentially expressed transcriptional regulators that are genetically required for limb bud and/or limb skeletal development (for details, see legend to Fig. 5B). This analysis revealed the expression profiles of these essential transcription factors in the different cell populations. For example, *Hoxa11*, *Hoxa13* and the *Hoxd11-13* genes were expressed at higher than average levels in S9^−^JAG1^+^ LMPs, as expected from their expression in the posterior-distal limb bud mesenchyme (left lane, Fig. 5B; reviewed by Zakany and Duboule, 2007). These Hox genes were also expressed at higher levels in S9^+^Pα^hi^*Col2a1*^+^ chondroblasts, in agreement with their requirement for limb skeletal bone development (right lane, Fig. 5B; González-Martín et al., 2014; Neufeld et al., 2014). Other than that, the signature of S9^−^JAG1^+^ LMPs was largely complementary to one of S9^+^Pα^hi^ OCPs and S9^+^Pα^hi^*Col2a1*^+^ chondroblasts (Fig. 5B). The S9^−^Pα^hi^ LMP population expressed higher than average levels of transcription factors functioning the anterior, posterior and/or peripheral mesenchyme during early limb bud development, such as *Pax1*, *Alx4*, *Irx3/5*, *Tbx2/3* and *Msx1* (second lane in Fig. 5B), which confirmed that this population is distinct from S9^−^JAG1^+^ LMPs. As expected, S9^+^Pα^hi^*Col2a1*^+^ chondroblasts expressed high levels of transcriptional regulators required for chondrogenesis and for limb skeletal, digit and tendon morphogenesis, such as Hoxa and Hoxd, Sox and Runx gene family members, and the *Shox2*, *Osr1* and *Scx* transcription factor genes (right lane in Fig. 5B).

Next, we assessed the chondrogenic differentiation potential of the two LMP populations identified in high-density culture (Fig. 5C; Barna and Niswander, 2007; Benazet et al., 2012). This resulted in activation of *Sox9*-EGFP expression in a significant fraction of cells from both LMP populations within 24 h, as previously reported for SOX9-negative digit progenitors isolated at much later stages (E11.5; Raspopovic et al., 2014). Moreover, the SOX9-positive cells in these cultures aggregated and formed typical chondrogenic condensations without addition of exogenous BMP4 (Fig. 5C). This analysis revealed the potent chondrogenic differentiation potential of both the S9^−^JAG1^+^ and S9^−^Pα^hi^ LMP populations, which together with the gene expression analysis (Figs 4 and 5A,B) indicated that they are likely progenitors of the chondrogenic lineage in forelimb buds.

### Differential cellular responsiveness uncovers the specific requirement of SHH and FGF signaling for S9^−^JAG1^+^ and S9^−^Pα^hi^ LMPs in culture

Limb bud outgrowth and patterning are controlled by the self-regulatory SHH/GREM1/AER-FGF feedback signaling system (reviewed by Zuniga, 2015). The transcriptome datasets of the four cell populations with chondrogenic differentiation potential (Figs 4 and 5) allowed us to analyze the differential expression of genes functioning in these pathways in an unbiased and genome-wide manner. This showed that the expression of genes functioning in SHH signal transduction by smoothened (SMO) was very different among the four mesenchymal cell populations (Fig. 6A). In particular, the SMO gene expression signature in S9^−^JAG1^+^ LMPs in the posterior-distal mesenchyme and S9^+^Pα^hi^ OCPs in the core mesenchyme appeared rather complementary (Fig. 6A, compare to Fig. 2A,C). The genes expressed at high levels in S9^+^Pα^hi^ OCPs (and in chondroblasts at E11.5) included direct transcriptional targets that are negatively regulated by SHH signaling and expressed predominantly in the core mesenchyme (e.g. *Cdon*, *Boc*, *Gli2* and *Hhip*; Tenzen et al., 2006; Probst et al., 2011; Lewandowski et al., 2015). By contrast, S9^−^JAG1^+^ LMPs expressed high levels of genes such as *Gli1* and *Ptch1*, which are activated in the posterior mesenchyme in response to SHH signaling (Goodrich et al., 1996; Büscher and Ruther, 1998). As this pointed to potential differences in the SMO-mediated response to SHH signaling, we treated forelimb bud mesenchymal cells (E10.5-E10.75) in high-density culture with cyclopamine, a SMO small-molecule antagonist (Chen et al., 2002). Twelve hours of cyclopamine treatment caused loss of *Gli1* expression, a direct transcriptional target of SHH-mediated signal transduction (Fig. 6B and Fig. S4A; Lee et al., 1997). Importantly, this relatively short cyclopamine treatment did not alter cell survival but slightly decreased the fraction of mitotic cells (Fig. S4B,C). Comparative flow cytometric analysis of control and cyclopamine-treated cultures revealed a significant reduction in both the S9^−^JAG1^+^ (∼3-fold) and S9^−^Pα^hi^ LMP populations (∼2-fold; Fig. 6B), while the large fraction of S9^+^Pα^hi^ OCPs was not altered by inhibiting SHH signal transduction (Fig. 6B). These results showed that maintenance of the two LMP populations in culture depended crucially on SHH signal transduction. As S9^−^JAG1^+^ LMPs are located in the posterior-distal mesenchyme close to the SHH source (Fig. 2C), we wondered whether these LMPs include *Shh-*expressing cells and/or their descendants (Harfe et al., 2004). The *Shh*^GFPCre^ allele, which labels *Shh*-expressing cells by EGFP (first panel in Fig. 6C) was used in combination with a CRE-inducible *ROSA26*^LSL−tdTomato^ transgene to trace the tdTOMATO-positive *Shh* descendants (second panel in Fig. 6C; Harfe et al., 2004). This approach identified a small fraction of cells expressing both tdTOMATO and JAG1 (fourth panel in Fig. 6C). This was also confirmed by FACS as ∼10% of the tdTOMATO^+^ LMPs co-expressed JAG1 (Fig. 6D). Therefore, it appears that only a small fraction of S9^−^JAG1^+^ LMPs originated from *Shh*-expressing cells and/or their descendants, pointing to the cellular heterogeneity of this population.

AER-FGF signaling is required to maintain cells in the distal sub-AER mesenchyme in an undifferentiated and proliferative state (ten Berge et al., 2008), which suggested that it might be required to maintain/expand LMPs in culture. Indeed, S9^−^JAG1^+^ LMPs express the highest levels of direct transcriptional targets of FGF signal transduction in the limb bud mesenchyme (*Spry1*, *Spry 2*, *Spry 4* and *Dusp6*, Fig. S5A; Kawakami et al., 2003; Morgani et al., 2018). As FGF8 is the main AER-FGF (Lewandoski et al., 2000), we assessed the effects of treating forelimb bud mesenchymal cells in culture with FGF8b for 12 h. As expected, the FGF8b treatment did not alter cell survival, but increased the fraction of cells in S phase and the expression of the direct targets *Spry4* and *Dusp6* (Fig. S5B-D). Flow cytometric analysis revealed that FGF8b treatment increased the fraction of S9^−^JAG1^+^ LMPs by ∼2-fold, while the S9^−^Pα^hi^ LMP population remained constant and the fraction of S9^+^Pα^hi^ OCPs was slightly reduced (Fig. S5D). Together, this analysis provided experimental evidence that S9^−^JAG1^+^ LMPs isolated from early limb buds depend most crucially on SHH and FGF signaling in high-density cultures (Fig. 6 and Fig. S5).

### GREM1-mediated BMP antagonism protects the immature S9^−^JAG1^+^ LMPs from precocious BMP-induced apoptosis

The majority of genes associated with GO term ‘cellular response to BMP signaling’ were expressed at lower than average levels in S9^−^JAG1^+^ and S9^−^Pα^hi^ LMPs (Fig. 7A). However, genes expressed at high levels by S9^−^JAG1^+^ LMPs included the BMP antagonist *Grem1*, which is expressed by a fraction of the *Jag1*-positive limb bud mesenchyme (Panman et al., 2006). Expression of several other BMP pathway genes was also increased in S9^−^JAG1^+^ LMPs, such as *Msx1*, *Bmp4*, *Bmp2* and *T* (brachyury), which are normally expressed in the posterior and/or distal limb bud mesenchyme (Catron et al., 1996; Liu et al., 2003; Bandyopadhyay et al., 2006; Benazet et al., 2009). S9^−^Pα^hi^ LMPs also expressed higher levels of *Msx1*, *Msx2* and *Bmp4*, but not *Grem1*, which showed that this population does not overlap the *Grem1*-expression domain in limb buds (Fig. 7A). This global analysis not only revealed distinct molecular differences between S9^−^JAG1^+^ and S9^−^Pα^hi^ LMPs populations, but also highlighted the expression of BMP response genes that function in chondrogenesis in S9^+^Pα^hi^ OCPs. The higher expression of *Col2a1* transcripts in S9^+^Pα^hi^ OCPs suggested that a fraction of them already initiated chondrogenic differentiation in forelimb buds at E10.5-E10.75 (Fig. 7A, compare with Fig. 3C). However, direct comparison of BMP response genes showed that S9^+^Pα^hi^*Col2a1*^+^ chondroblasts at E11.5 expressed higher levels of genes that function in chondroblast differentiation and maturation than S9^+^Pα^hi^ OCPs, such as *Acan*, *Col2a1*, *Chrdl1*. *Bmpr1*, *Nog* and *Adamts12* (Fig. 7A).

Unexpectedly, these results indicated that the SOX9-positive OCPs located in the core mesenchyme were already exposed to higher BMP activity in early forelimb buds than the SOX9-negative LMPs in the peripheral and posterior-distal mesenchyme. To assess their response to BMP signaling, LMPs and OCPs were cultured in medium containing BMP4 for 24 h (Fig. 7B). While S9^+^Pα^hi^ OCPs activated COL2A and formed aggregates typical of chondrogenic condensations (lower panels, Fig. 7B), no COL2A and fewer to no SOX9-positive aggregates were detected in S9^−^JAG1^+^ and S9^−^Pα^hi^ LMP cultures (upper and middle panels, Fig. 7B). In fact, overall cell numbers decreased (Fig. 7B), which prompted us to assess BMP-induced apoptosis (Fig. 7C). Indeed, the apoptosis of both LMP populations increased significantly, while the survival of S9^+^Pα^hi^ OCPs was not altered. Next, the effects of inhibiting BMP signal transduction on the different cell populations were assessed. Dorsomorphin, a selective inhibitor of BMP type I receptors (Yu and Ornitz, 2008), reduced BMP signal transduction in unsorted mesenchymal cell cultures (E10.5-E10.75) by 40-50% within 12 h (Fig. S6A) and increased overall apoptosis by ∼2-fold (left panel in Fig. 7D). However, among the live Lin^−^ cells, the S9^−^JAG1^+^ and S9^−^Pα^hi^ LMPs increased by ∼4 to ∼7-fold, respectively, while the fraction of S9^+^Pα^hi^ OCPs was reduced (Fig. 7D). Together, these results (Fig. 7B-D) indicated that lower BMP levels favor LMPs in high-density cultures.

The proposed protective role of BMP antagonism for LMPs was genetically assessed by flow cytometric analysis of forelimb buds from wild-type and *Grem1*-deficient littermate embryos (E10.5-E10.75). Owing to the complexity of analysis and small numbers of cells recovered, overall cell death was ∼2-fold higher than normal even in wild-type controls (Fig. S6B, see Materials and Methods). Among the live Lin^−^ mesenchymal cells, the fraction of S9^−^JAG1^+^ LMPs was reduced by ∼42%, while S9^−^Pα^hi^ LMPs were not significantly affected and the fraction of the predominant S9^+^Pα^hi^ OCPs increased by ∼15% in *Grem1*-deficient forelimb buds (Fig. 6E). These results show that GREM1-mediated BMP antagonism (Zuniga et al., 1999) preferentially impacts the immature S9^−^JAG1^+^ LMPs located in the distal-posterior forelimb bud mesenchyme. In particular, this analysis highlighted the importance of GREM1-mediated protection of LMPs from premature exposure to BMP signaling in early forelimb buds. At the same stage, the OPCs located in the core mesenchyme already express higher levels of BMP target genes that function in the onset of chondrogenesis. This reveals the differential exposure and response of the core and peripheral/posterior-distal mesenchyme to BMP signaling and GREM1-mediated antagonism in early forelimb buds (E10.5-E10.75).

## DISCUSSION

In this study, we quantitate limb bud mesenchymal cell numbers and show that the proportion of mesenchymal cells in S phase is highest in early forelimb buds, whereas the fraction of LMPs in G0/G1 increases during distal progression of outgrowth. These results corroborate one of our previous studies, which showed that GLI3 promotes the BMP-dependent cell cycle exit of digit progenitors during initiation of chondrogenic differentiation (Lopez-Rios et al., 2012). Studies by others have shown that distal progression of limb bud outgrowth also depends on oriented divisions of the limb bud mesenchymal cell and cell shape changes (Boehm et al., 2010; Gros et al., 2010). To gain insight into the cellular heterogeneity of mesenchymal progenitors during outgrowth and patterning, we combined cell sorting with RNA-seq analysis of forelimb buds at E10.5-E10.75. This analysis identified three distinct SOX9-negative mesenchymal progenitor cell populations in addition to SOX9-positive OCPs in early forelimb buds. Our transcriptome analysis reveals both the population-specific gene expression signatures and the distinct transcriptional responses of the different mesenchymal cell populations to SHH and BMP signaling in early forelimb buds. This analysis also identified S9^−^SCA-1^+^ mesenchymal cell population as the one encompassing the myogenic progenitors migrating into forelimb buds (reviewed by Francis-West et al., 2003; Epting et al., 2004). As these S9^−^SCA-1^+^ cells also express PDGFRα, it is important to note that this FACS signature does not appear to be enriched in Pα^+^SCA-1^+^ MSCs in forelimb buds (E10.5-E10.75) in contrast to developing limb long bones (Morikawa et al., 2009; Nusspaumer et al., 2017).

The transcriptome analysis, together with their differentiation potential in culture, provides evidence that S9^−^JAG1^+^ LMPs and S9^−^Pα^hi^ LMPs are early progenitors with significant chondrogenic differentiation potential. The S9^−^JAG1^+^ LMPs express markers of the posterior-distal mesenchyme in proximity to the source of SHH signaling, whereas S9^−^Pα^hi^ LMPs express a more diverse set of markers of anterior, posterior and distal mesenchyme. This contrasts with the transcriptional signature of the S9^+^Pα^hi^ OCPs that reside in the core mesenchyme and give rise to chondroblasts. Previous analysis of more-advanced limb buds (E11.5) showed that WNT and FGF signals emanating from the ectoderm and AER keep the underlying distal mesenchyme in a proliferative and undifferentiated state (ten Berge et al., 2008; Gros et al., 2010). This is corroborated by our analysis, which shows that FGF8b increases the fraction of cells in S-phase. FGF8b treatment also specifically increases the fraction of S9^−^JAG1^+^ LMPs in high-density culture. By contrast, the abundance of S9^−^Pα^hi^ LMPs is not altered in response to FGF signaling. In agreement, our transcriptome analysis revealed that the S9^−^JAG1^+^ LMPs located in the posterior-distal mesenchyme express highest levels of FGF target genes (Kawakami et al., 2003; Morgani et al., 2018). During the onset of limb bud development, SHH signaling specifies antero-posterior digit identities and subsequently promotes the proliferative expansion of mesenchymal progenitors (Towers et al., 2008; Zhu et al., 2008). Our analysis shows that S9^−^JAG1^+^ LMPs express the highest levels of target genes functioning in the positive response to SHH signal transduction, such as *Gli1* and *Ptch1* (reviewed by Lopez-Rios, 2016). In contrast, the OCPs located in the core mesenchyme express high levels of genes (e.g. *Cdon*, *Boc* and *Hhip*) that are negatively regulated by SHH signal transduction (Tenzen et al., 2006; Probst et al., 2011; Lewandowski et al., 2015). Inhibition of SHH signal transduction in culture shows that S9^−^JAG1^+^ and S9^−^Pα^hi^ LMPs, but not S9^+^Pα^hi^ OCPs, depend crucially on SHH signaling. As no significant changes in overall mesenchymal cell cycle kinetics and apoptosis were detected after 12 h treatment, the reduction in the two LMP populations might reflect changes in their fates and/or be the result of them undergoing differentiation. Both LMP populations also express high levels of *Mycn* (also known as *N-Myc*) which regulates limb bud mesenchymal cell proliferation (ten Berge et al., 2008; Towers et al., 2008; this study). It has been shown that smaller condensations and skeletal elements form in *Mycn*-deficient mouse limbs as a consequence of the premature depletion of mesenchymal progenitors (Ota et al., 2007). Additional cell cycle regulators expressed in the distal limb bud mesenchyme include *Cdk6*, which regulates cell cycle progression, and its inhibitor *Cdkn2c* (Lopez-Rios et al., 2012; Lewandowski et al., 2015). Interestingly, S9^−^JAG1^+^ LMPs express the highest levels of *Cdk6*, while *Cdkn2c* is expressed by the other cell populations analyzed (this study).

The balance between proliferative expansion of LMPs and their exit toward chondrogenic differentiation is controlled by the GLI3 repressor, which regulates both the cell cycle and *Grem1*-mediated BMP antagonism (Lopez-Rios et al., 2012). To transit from SOX9-negative LMPs to SOX9-positive OCPs and chondrogenic differentiation, the mesenchymal progenitors switch from responding to growth-promoting signals to increased BMP activity (Benazet et al., 2012; Lopez-Rios et al., 2012). We show that, in early forelimb buds, the S9^+^Pα^hi^ OCPs located in the core mesenchyme are already exposed to higher BMP signal transduction. Furthermore, the proportion of S9^+^Pα^hi^ OCPs is increased in *Grem1*-deficient forelimb buds as a likely direct consequence of the increase in BMP activity. Of the two LMP populations analyzed, S9^−^Pα^hi^ LMPs express the highest levels of *Tbx2*, which encodes a transcriptional regulator that participates in repressing *Grem1* in the limb bud mesenchyme (Farin et al., 2013). This indicates that S9^−^Pα^hi^ LMPs could be in a transitory phase from immature LMPs towards OCPs. Indeed, *Grem1* is expressed only by the immature S9^−^JAG1^+^ LMPs, which are drastically reduced in number in *Grem1*-deficient forelimb buds. In fact, previous genetic analysis has shown that *Grem1* inactivation induces limb bud mesenchymal apoptosis due to precociously increased BMP activity (Bastida et al., 2004; Michos et al., 2004). This, together with our analysis, indicates that the BMP antagonist GREM1 protects the uncommitted and proliferating S9^−^JAG1^+^ LMPs from premature exposure to high BMP activity. As *Grem1* is expressed only by dorsal and ventral mesenchymal cells within the posterior-distal JAG1 domain (Panman et al., 2006), the extracellular GREM1 antagonist likely protects the non-expressing S9^−^JAG1^+^ LMPs in a paracrine manner. Others have reported a similar protective effect of the BMP antagonist noggin during joint development (Ray et al., 2015; Huang et al., 2016). Similarly, BMP activity is transiently reduced during regeneration of the tracheal epithelium by follistatin- and noggin-mediated BMP antagonism, thereby enabling epithelial self-renewal (Chung et al., 2018). Together, these studies and our study reveal a general protective function of BMP antagonists in averting premature and deleterious exposure of progenitor and stem cells to BMP signaling.

Our study establishes that the mesenchyme of early mouse forelimb buds is already composed of different types of progenitors with heterochronic cell specification and differentiation states. In particular, the immature S9^−^JAG1^+^ LMP population appears to crucially depend on SHH, AER-FGF signaling and *Grem1*-mediated BMP antagonism as part of the self-regulatory signaling system that coordinately controls limb bud patterning and outgrowth (reviewed by Zuniga, 2015). At the same early stage, the S9^+^Pα^hi^ OCPs located in the core mesenchyme already positively respond to BMP signal transduction. Our study provides insights into how this differential responsiveness coordinately regulates both the expansion and differentiation of OCPs in the core and immature S9^−^JAG1^+^ LMPs in the posterior-distal mesenchyme during the early phase of mouse forelimb bud development.

## MATERIALS AND METHODS

### Mouse strains and ethics statement

The *Prx1*-Cre (Logan et al., 2002), *b-ACTIN-loxP-stop-loxP-EGFP* (Jägle et al., 2007), *Sox9*^IRES−EGFP^ (Chan et al., 2011), *Shh*^GFPCre^ (Harfe et al., 2004), *ROSA26*^LSL−tdTomato^ (Madisen et al., 2010) and two *Grem1* loss-of-function alleles (delta and Del C alleles; Michos et al., 2004; Zuniga et al., 2004) were kept in a mixed Swiss Albino genetic background. Swiss Albino mice were purchased from Janvier. All experiments were performed on embryos strictly adhering to Swiss law, the 3R principles and the Basel Declaration. All animal studies were evaluated and approved by the Regional Commission on Animal Experimentation (license 1950 and 1951). Embryos of both genders were used at the indicated developmental stages.

### Quantitation of limb bud mesenchymal cell numbers

Dissected limb buds were digested in 1 ml 1×HBSS (Gibco) containing 1 mg/ml collagenase D and 50 µg/ml DNase I (Roche) at 37°C in FACS tubes. Single cells were counted by flow cytometry in defined sub-fraction volumes that were calibrated using TrueCount tubes with polystyrene fluorescent beads (BD Biosciences). The beads were gated in the GFP and propidium iodide (PI) channels. In order to calculate the volume acquired by the FACS in one minute, beads and/or cells from one limb bud were resuspended in 2 ml PBS and counted using a constant flow. This was repeated several times to assure that the volume calibrations and volume measurements were accurate. The volume fraction analyzed per minute was calculated as follows: V=counted beads/total beads (corresponding to the volume fraction per minute). Total cell numbers (C) were calculated as follows: C=(counted cells/V)×2000 [2000 (µl)=total volume used to resuspend either the cells from one limb bud or beads for calibration].

The GFP-positive LMPs correspond to the cells in which the *ßact*^GFP^ transgene has been activated by *Prx1*-Cre-mediated recombination. In contrast, non-limb bud mesenchymal cells and ectodermal cells do not express GFP. Gating of apoptotic cells showed maximally 8-12% of cell death during preparation of single cells.

### Cell cycle analysis by FACS

Forelimb buds were dissected, pooled (∼25 at E9.75, eight at E10.75 and six at E11.75) and dissociated using collagenase D. To remove ectodermal, endothelial and hematopoietic, and apoptotic cells by FACS, the cells were stained with a mix of biotinylated antibodies (EpCAM, Biolegend, clone G88; CD31, eBioscience, clone 390; TER119, Biolegend; CD45, Biolegend clone 30F11; CD11b, Biolegend clone M1-70; Gr1, Biolegend clone RB6-8C5) as previously described by Nusspaumer et al. (2017). Apoptotic cells were identified by double staining for Annexin V (APC-conjugated, Biolegend) and 7AAD. Following this combined staining of lineage-positive cells, all cells were fixed in 70% ethanol at −20°C for minimally 2 h. Phospho-histone H3 antibodies (BD Biosciences, clone HTA28 Alexa Fluor 647) were used to detect mitotic cells. Cells were also incubated with 50 µl/ml propidium iodide (Sigma) and 50 µl/ml RNAse A (Sigma) to measure their DNA content directly. For cell cycle analysis of cultured cells, mesenchymal cells were pooled from six forelimb buds at E10.5. For each embryo, one forelimb bud was used as untreated control and one for the experimental treatment. FACS was used to determine the fractions of cells in the different phases of the cell cycle and the fraction of phospho-histone H3-positive mitotic cells among the lineage-negative (Lin^−^) limb bud mesenchymal cells. To study the cell cycle by BrdU incorporation, pregnant mice were injected intra-peritoneally with 1 mg of BrdU (5 mg/ml in PBS, Sigma) 4 and 2 hours before analysis. Single cells prepared from 20 forelimb buds were analyzed. The BrdU-positive cells were detected using the APC BrdU Flow kit (BD Biosciences). FACS analysis was carried out using a BD FACSAria III machine. After exclusion of apoptotic and lineage-positive cells, the numbers of cells in different phases of the cell cycle, mitotic and BrdU-positive cells were determined and fractions calculated using the FlowJo 10.5.3 software.

### Immunofluorescence analysis

After fixation in 4% paraformaldehyde for 2 h at 4°C, limb buds were dehydrated. Then they were mounted in 50:50 (v/v) OCT/30% sucrose and 10 μm cryosections for immunofluorescence analyses prepared. Sections were permeabilized using PBS containing 0.2% Triton X-100 for 30 min at room temperature.

For immunofluorescence analysis of FACS-sorted LMP populations, cells were resuspended in complete DMEM/F12 medium (supplemented with 1% penicillin/streptomycin and 10% FBS, Merck) and 88,000 cells were seeded in one well of a 384-well plate (BD Biosciences). After culture, cells were fixed in 4% paraformaldehyde for 30 min at room temperature and then permeabilized as described above. Primary antibodies against the following proteins were used for immunofluorescence: GFP (1:250; 4745-105, Bio-Rad), jagged 1 (1:50; TS1.15H, Developmental Studies Hybridoma Bank), PDGFRα (1:250; AF1062, R&D), DsRed (1:1000; 632496, Clontech), SOX9 (1:10000; AB5535, Millipore) and COLII (1:250; MS-235-P1, Thermo Fisher Scientific). Signals were detected using the following fluorochrome-coupled secondary antibodies (1:250): anti-sheep Alexa Fluor 488 (713-545-147, Jackson ImmunoResearch), anti-mouse Alexa Fluor 594 (R37121, Thermo Fisher Scientific), anti-rabbit Alexa Fluor 594 (406418, Biolegend), anti-rat Alexa Fluor 647 (A-21247, Thermo Fisher Scientific) and anti-goat Alexa Fluor 647 (A-21447, Thermo Fisher Scientific). Nuclei were counterstained with Hoechst-33258. Images were captured using a Nikon Ti-E microscope equipped with Hamamatsu Flash 4.0 V2 CMOS camera, Yokogawa Spinning Disk CSU-W1-T2 and the VisiView Premier Image acquisition software. Pseudo-colors were chosen from the available lookup tables.

### RNA whole-mount *in situ* hybridization

Whole-mount *in situ* hybridization was carried out as described previously (Haramis et al., 1995).

### FACS isolation of mouse LMP populations

Single cell suspensions were prepared from 60 to 160 mouse embryonic forelimb buds at E10.5-E10.75 (35-38 somites) and E11.5 (46-48 somites; for chondroblasts only). Dissected limb buds were collected into ice-cold PBS and digested for up to 15 min in 1 mg/ml collagenase D in high glucose DMEM medium at 37°C. Limb buds were gently pipetted every 5 min until the tissue was dissociated into single cells. Ice-cold HBSS supplemented with 2% FBS and 10 mM HEPES was added to stop digestion. The cell suspensions were filtered to remove aggregates. During FACS analysis, the lineage-positive ectodermal, endothelial and hematopoietic cells (see above), and apoptotic cells were excluded by gating. Apoptotic cells were detected using 7AAD (Biolegend) and in general amounted to ∼20-30% of all mesenchymal cells at E10.5-E10.75. After the initial gating, the lineage-negative (Lin^−^) cells were separated into different populations using the following antibodies: anti-PDGFRα (CD140a; clone APA5: BV421-conjugated, Biolegend); anti-JAG1 (clone HMJ1-29: PE-conjugated, Biolegend) and anti-SCA-1 (clone D7: APC-conjugated, eBioscience). Streptavidin was conjugated to APC/Cy7. Cells were sorted using a FACSAria III (BD Bioscience) equipped with an 85 μm nozzle in combination with the FACS Diva software V8.0. After sorting, the different cell populations were re-analyzed to assess their viability and purity. FACS plots were generated using the FlowJo 10.5.3 and GraphPad Prism 7 software. Bar graphs, and box and whisker plots were generated using GraphPad Prism 7.

### RNA-seq analysis

Thirty to 80 forelimb buds were collected from *Sox9*^IRES−EGFP^ embryos at E10.5-E10.75 to purify the different mesenchymal cell populations. S9^+^Pα^hi^*Col2a1*^+^ chondroblasts were isolated from 12 to 30 forelimb buds at E11.5. RNA was extracted using the RNeasy Microkit (Qiagen) and the RNA quality determined using RNA 6000 Pico kit (Agilent 2100 Bioanalyzer). Only samples with an RNA integrity ≥8.5 were used. Libraries were prepared from 15 ng of total RNA after purification of poly(A)^+^ RNA using NEBNext kits according to manufacturer's instructions. Libraries were sequenced using the HiSeq 2500 Illumina sequencer with the single-read 50 cycles protocol. Single-end RNA-seq reads were mapped to the mouse genome mm10 assembly using RNA-STAR (Dobin et al., 2013). For reporting multi-mappers, only one hit in the final alignment files (outSAMmultNmax=1) was used and reads without evidence in splice junction tables were filtered out (outFilterType=‘BySJout’). Raw reads and the mapping quality were assessed using the qQCreport function of the R/Bioconductor software package QuasR (version 1.18.0; Gaidatzis et al., 2015). The RefSeq mRNA coordinates from UCSC (genome.ucsc.edu) and the qCount function from the QuasR package were used to quantify gene expression by the number of reads starting within any of the annotated exons of the gene of interest.

### Hierarchical clustering, heatmaps and statistical testing

The subsequent gene expression data analysis was carried out using R software (version 3.4.2, R Foundation for Statistical Computing) and the corresponding software packages of Bioconductor (version 3.6; Huber et al., 2015). Differentially expressed genes were identified using the edgeR package (version 3.20.1; Robinson et al., 2010). Genes with *P*≤0.1 and absolute log_2_ fold changes ≥1.2 were considered as differentially expressed. Principal component analysis was performed with log_2_ transformed CPM values using 25% of the most variable genes. Heatmaps show row-centered log2 transformed CPM values. The 1-Pearson correlation coefficient was used as distance measure for hierarchical clustering (‘complete’ method). In order to enhance the color scale, values outside the 0.05%-99.5% percentile range were replaced with the corresponding percentile value. MsigDb (v6.0, Broad Institute) was used in competitive gene set enrichment analysis. Human EntrezGene IDs were converted to mouse EntrezGene IDs using the HomoloGene database (NCBI, build 68). Only gene sets consisting of at least 10 genes were tested with the ‘camera’ function from edgeR package. A false discovery rate (FDR) of <0.05 was set as cut-off.

### Limb bud mesenchymal cell cultures

Forelimb buds from two mouse embryos (E10.5-E10.75) were incubated in ice-cold 2% trypsin (Gibco)/PBS at 4°C for 30 min and the digestion was stopped by an excess of DMEM medium with 10% fetal bovine serum (FBS). The limb bud ectoderm was manually removed and mesenchymal cells dissociated by gentle pipetting. Cells were plated in four wells of a 96-well plate in high-glucose DMEM medium (10% FBS, 4.5 g/l glucose, 100 U penicillin, 0.1 mg/ml streptomycin and 200 mM L-glutamine, Merck). After 8-9 h, non-adherent cells were removed by changing the medium. Two wells were treated with either 20 μM cyclopamine KAAD (dissolved in ethanol; Calbiochem), 300 ng/μl FGF8b (dissolved in PBS, 0.1%BSA; R&D), 5 μM dorsomorphin (dissolved in DMSO; Merck) or with 20 ng/ml BMP4 (dissolved 4 mM HCl; R&D) for 24 h, while others served as controls (normal medium with solvent). After 12 h, cells were gently detached using trypsin and either processed for FACS analysis of specific cell populations (see before) or processed for RT-qPCR analysis (see below). The Wilcoxon test was used to statistically verify differences observed.

### RT-qPCR analysis

After culture, cells were flash frozen in RLT buffer and RNA was isolated using Qiagen RNeasy Microkit. cDNA was prepared from 300 ng of total RNA that was quantified using the Qubit RNA HS assay. For each sample, the relative expression was normalized to the housekeeping gene *Rpl19* and to the target gene expression level in the untreated condition (ΔΔCt method). Transcripts detected with Ct≥32 were considered as non-expressed genes. To statistically verify significant differences in the relative gene expression, the Wilcoxon and Mann-Whitney tests were used. Details of the oligos used for gene expression analysis can be found in Table S11.

### FACS analysis of mesenchymal cells from *Grem1*-deficient mouse limb buds

Mice heterozygous for a *Grem1* loss-of-function allele were crossed to isolate littermate embryos of the different genotypes at E10.5-E10.75. Single cells were prepared from pairs of forelimb buds for each embryo and divided into two samples to assess both apoptosis and the fractions of S9^−^JAG-1^+^ and S9^−^Pα^hi^ LMPs and S9^+^Pα^hi^ OCPs by FACS, which allowed analysis of ∼70,000 cells per sample. This analysis was carried out blindly as the embryos were genotyped only after the FACS analysis of the cells was already complete. The longer processing times, together with the lower numbers of cells analyzed, explains the ∼2-fold increase in overall cell death as detected by APC-conjugated Annexin-V and 7AAD (Fig. S6B). Only a fraction of live and Lin^−^ limb bud mesenchymal cells was analyzed to determine the fractions of the three cell populations, which, together with sufficiently large numbers of biological replicates, resulted in significant and biological meaningful results that were statistically verified using the Mann-Whitney test.

## Supplementary Material

Supplementary information
