# Supplementary material for: Molecular signatures identify immature mesenchymal progenitors in early mouse limb buds that respond differentially to morphogen signaling
Source: Development. 2019 May 28;146(10):dev173328. doi: 10.1242/dev.173328 (PMC6550019; doi:10.1242/dev.173328)
Supplement: Supplementary information [file develop-146-173328-s1.pdf]

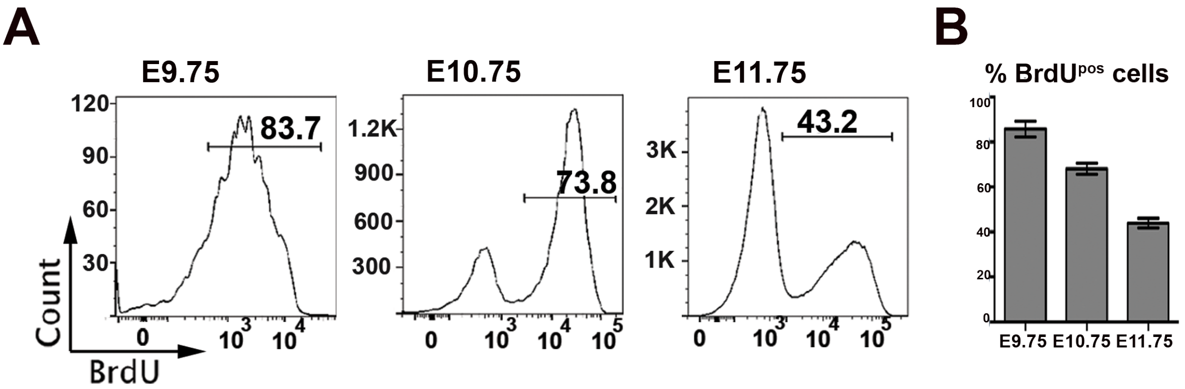

Fig. S1. Fraction of BrdU-positive mesenchymal cells at different forelimb bud stages.

(A) Representative FACS analysis shows the BrdU incorporation into wild-type forelimb buds at E9.75 (26-29 somites, n=5 independent samples), E10.75 (36-40 somites, n=4) and E11.75 (48-52 somites, n=5). Numbers indicate the percentage of BrdU-positive cells. (B) Percentage of BrdU-positive cells in wild-type forelimb buds (E9.75: n=5, E10.75: n=4 and E11.75: n=5 independent samples).

Development • Supplementary information

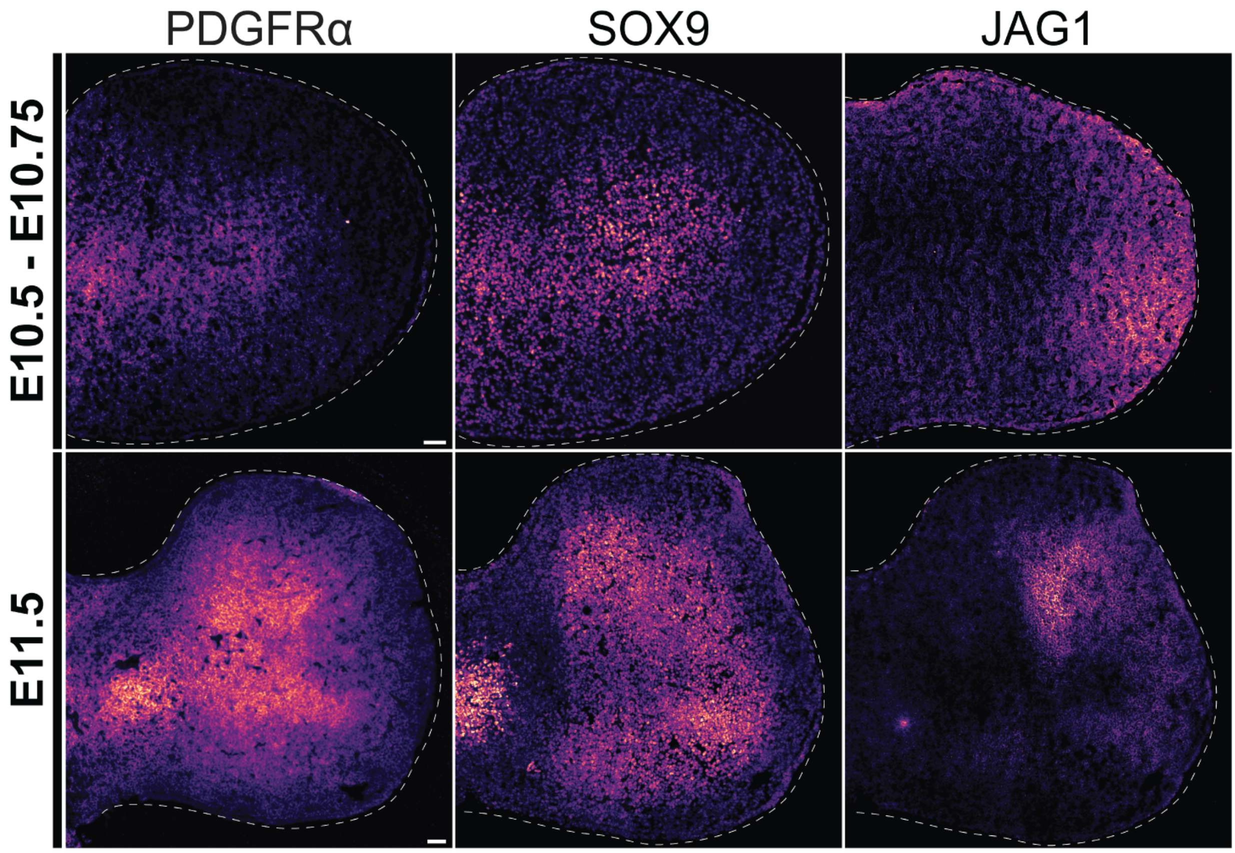

Fig. S2. Spatial distribution of markers used to identify specific mesenchymal cell populations in forelimb buds.

Immunohistochemistry shows the spatial distribution of the SOX9, PDGFRα and JAG1 proteins in mid-sagital sections of mouse forelimb buds at E10.5 and E11.5. Note that the mesenchymal cells expressing JAG1 at E11.5 overlap with SOX9-positive cells in the anterior mesenchyme. This was confirmed by FACS analysis. Therefore, JAG1 is only marking the posterior-distal and SOX9-negative mesenchymal cells in early forelimb buds at E10.5 (see also Fig. 2). White dashed lines outline limb bud. Scale bars: 50 μm.

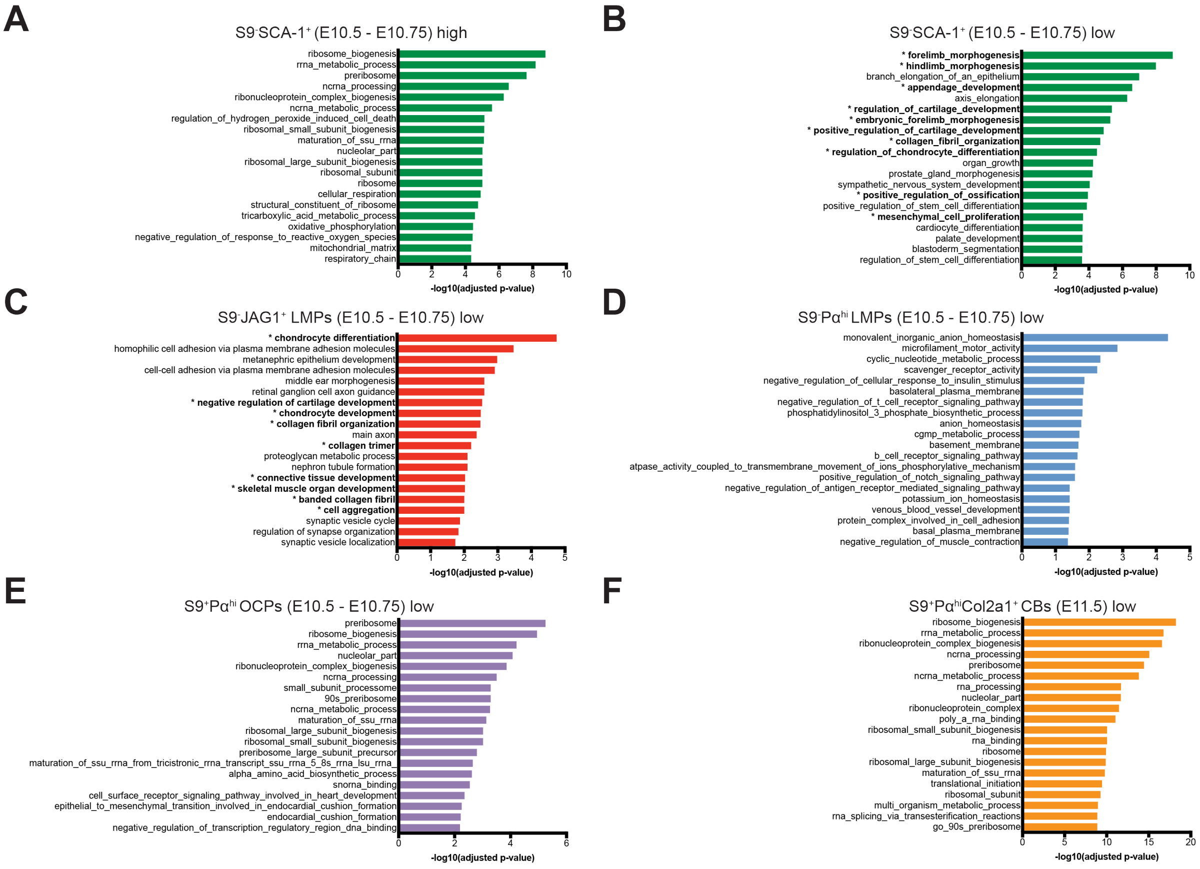

Figure S3. GO analysis of the genes expressed differentially in the forelimb bud mesenchymal cell populations at E10.5-E10.75.

(A, B) GO analysis of the genes whose expression is higher (panel A) and lower than average (panel B) in the S9$^-$SCA-1$^+$ mesenchymal cell population. (C-F) GO analysis of genes expressed at lower than average levels in S9$^-$JAG1$^+$ LMPs (panel C), S9$^-$Pα^{hi} LMPs (panel D), S9$^+$Pα^{hi} OCPs (panel E) and S9$^+$Pα^{hi}*Col2a1*$^+$ chondroblasts (panel F). Asterisks indicate chondrogenesis- and limb-related GO terms.

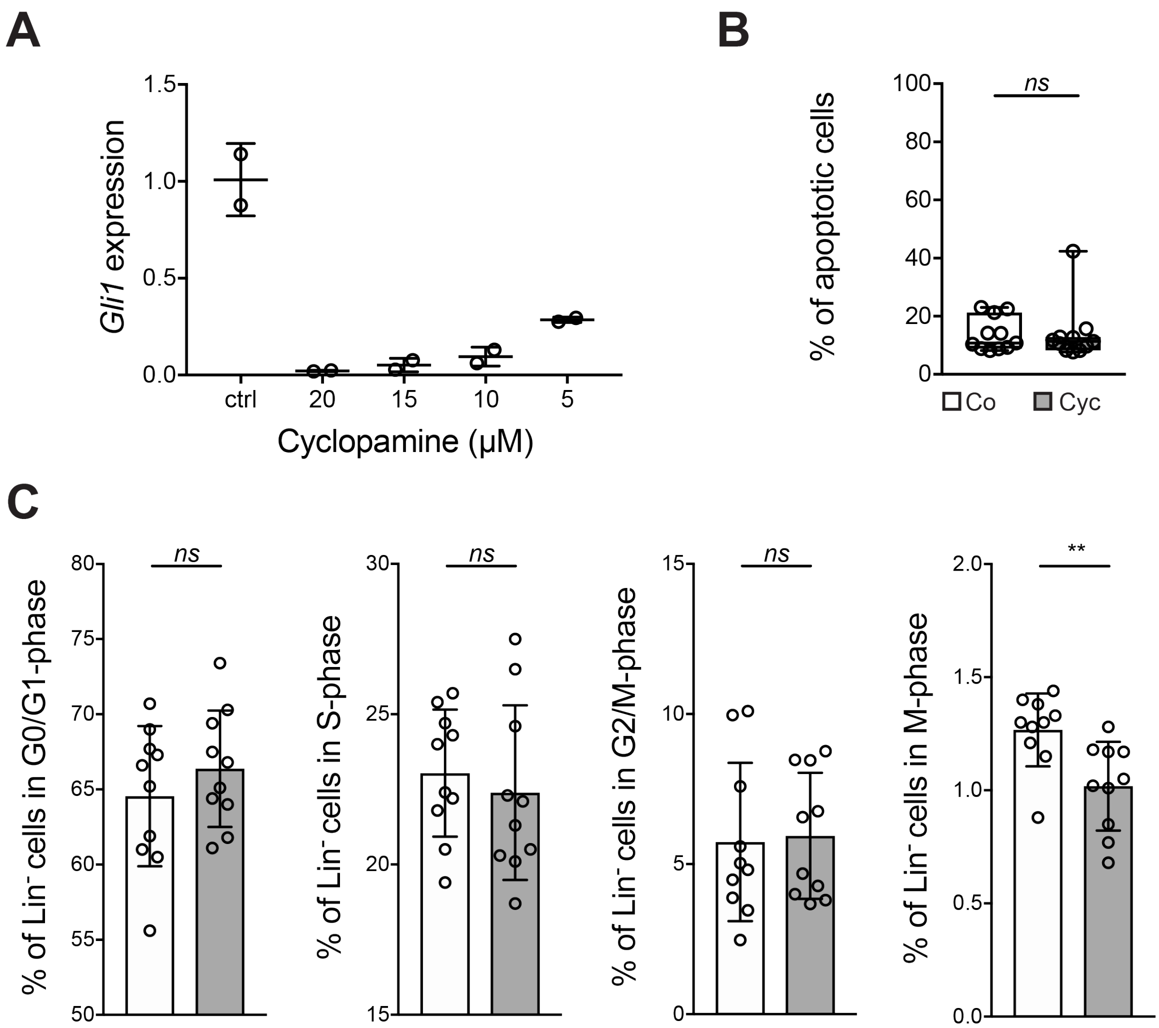

Fig. S4. SHH pathway analysis.

(A)Limb mesenchymal cells were cultured for 12 hours in presence of different concentrations of cyclopamine (0-20µM). Graph showing relative *Gli1* expression levels as determined by RT-qPCR. Individual data points plus mean ± SD are shown (n=2 data points per concentration). (B) Apoptosis rate assessed by Annexin-V in lineage-negative limb bud culture cells treated with 20µM cyclopamine (Cyc) or solvent alone (Co). Individual data points plus mean ± SD are shown (n=11). (C) Quantification of cell cycle stages occupied by limb mesenchymal cells after 12 hours of cyclopamine treatment. Individual data points plus mean ± SD are shown (n=10). Statistical evaluation of all results was done using the Wilcoxon test: (**) p-value ≤0.01.

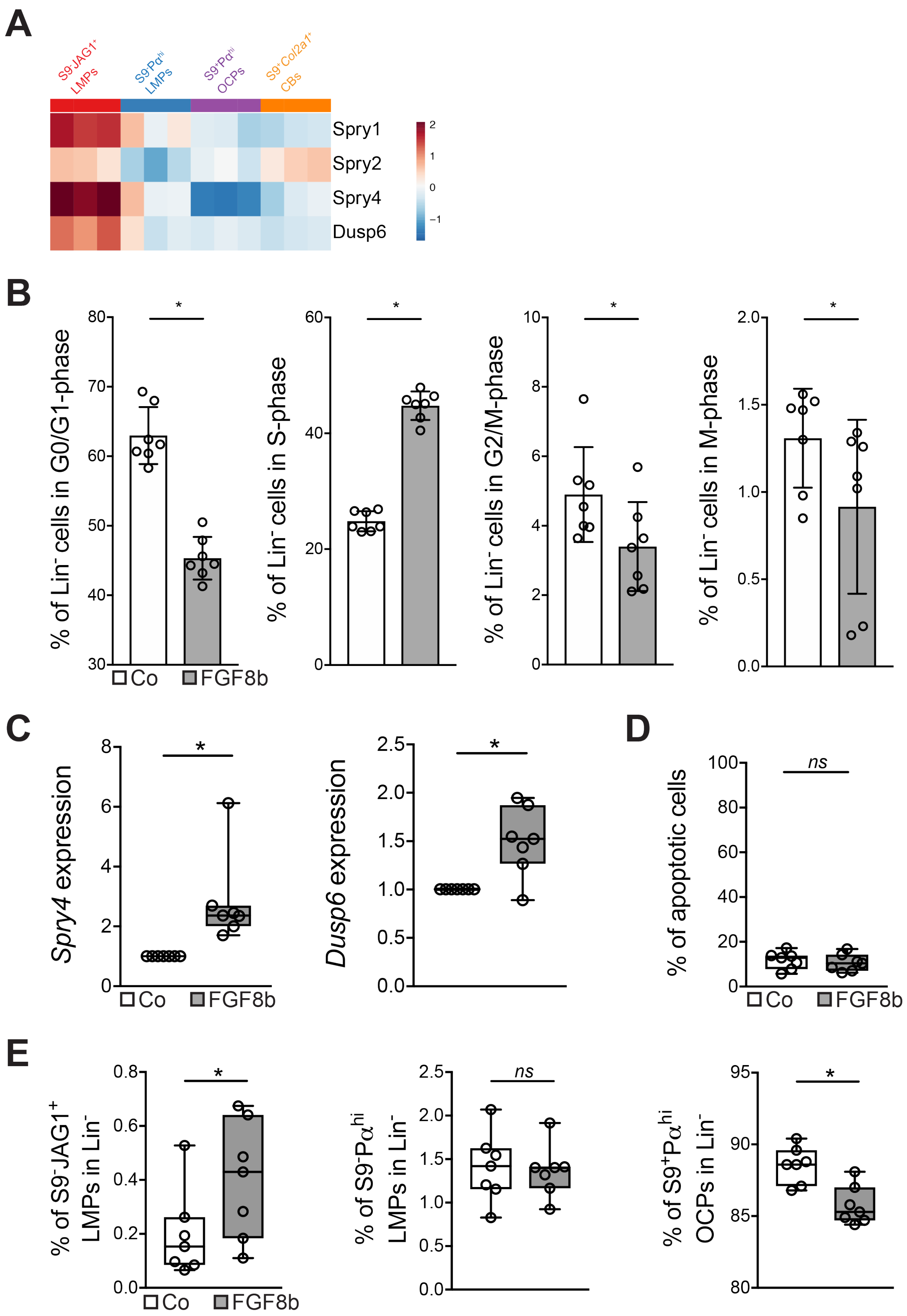

Development • Supplementary information

Fig. S5. FGF pathway analysis.

(A) $S9^{-}JAG1^{+}$LMPs express highest levels of the *Spry* and *Dusp6* transcriptional targets of FGF signaling in limb buds. (B) Forelimb bud mesenchymal cells (E10.5) were cultured for 12 hours in medium supplemented with FGF8b (300ng/mL) or solvent alone (Co). The fractions cells at the different stages of the cell cycle were quantitated by FACS. Individual data points plus mean ± SD are shown (n=7). (C) The effects of the FGF8b treatment on *Spry4* and *Dusp6* expression levels in cultured mesenchymal cells was determined by RT-qPCR (levels in control cultures were set arbitrary to 1). (D) Lin^{-} mesenchymal cells undergoing apoptosis in control and FGF8b-treated cultures. Individual data points plus mean ± SD are shown (n=7). (D) FACS quantitation of the different stages of the cell cycle in limb bud mesenchymal cells (controls versus FGF8b treated). Individual data points plus mean ± SD are shown (n=7). (E) Comparative analysis of the fractions (%) of $S9^{-}JAG1^{+}$ and $S9^{-}P\alpha^{hi}$ LMPs and $S9^{+}P\alpha^{hi}$ OCPs in control and FGF8b treated cultures. Statistical evaluation of all results was done using the Wilcoxon test: (*) p-value ≤0.05.

Development • Supplementary information

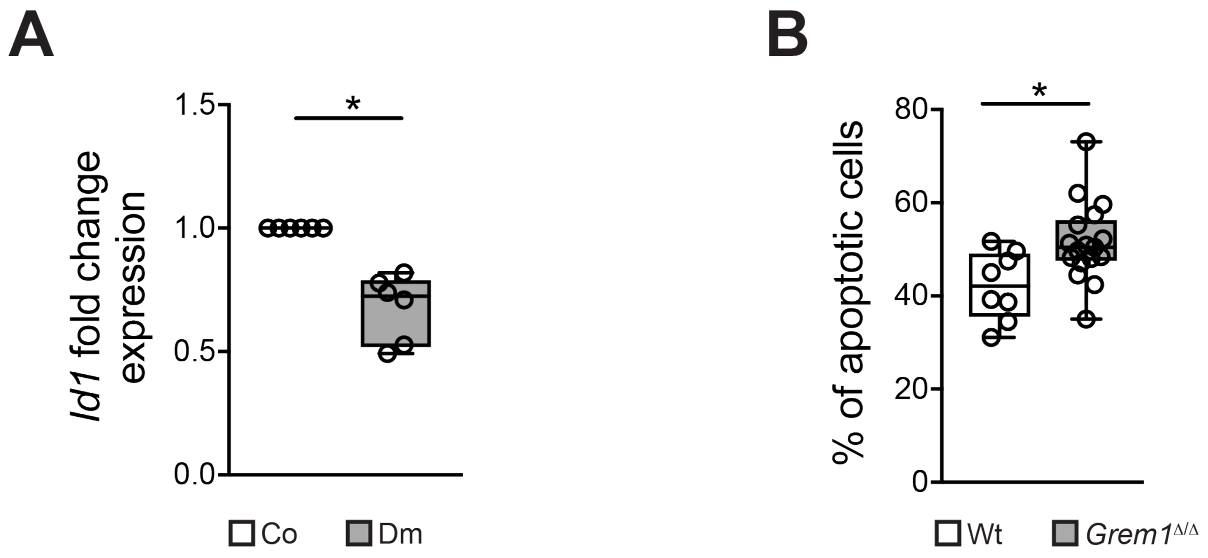

Fig. S6. BMP and *Grem1* pathway analysis.

(A) Limb mesenchymal cells (E10.5) were cultured for 12 hours in medium supplemented with solvent (Co) or 5 μM Dorsomorphin (Dm). This reduces the expression of the direct transcriptional target *Id1* as determined by RT-qPCR analysis. Individual data points plus mean ± SD are shown (n=6). (B) FACS was used to determine the fraction of apoptotic cells isolated from wild-type (Wt) and *Grem1*-deficient forelimb buds (*Grem1*$^{\Delta/\Delta}$) at E10.5. Individual data points plus mean ± SD are shown (n=8 for *Grem1*$^{\Delta/\Delta}$; n=17 for Wt). Statistical evaluation of all results was done using the Wilcoxon test: (*) p-value ≤0.05.

Development • Supplementary information

Table S1. Values myogenic-lineage-specific genes

Click here to Download Table S1

Table S2. Differentially expressed genes (DEGs) Sca-1 population

Click here to Download Table S2

Table S3. DEGs Jag1

Click here to Download Table S3

Table S4. DEGs PDGFRa

Click here to Download Table S4

Table S5. DEGs OCPs

Click here to Download Table S5

Table S6. DEGs chondroblasts

Click here to Download Table S6

Table S7. Data for the switch-peak heatmap

Click here to Download Table S7

Table S8. Data for "Smoothened (SMO) signaling pathway" (GO:0007224)

Click here to Download Table S8

Table S9. Data for "Cellular response to BMP stimulus" (GO:0071773)

Click here to Download Table S9

Table S10. Data for manually curated list of transcription factors with essential functions during limb development (subset of Table S7)

Click here to Download Table S10

Table S11. The oligos used for gene expression analysis

Acan fwd: 5′-AGTCAACCGTTGCAGACCAG-3′

Acan rev: 5′-GGTCATGAAAGTGGCGGTAA-3′

BMP4 fwd: 5′-AGCCGAGCCAACACTGTGA-3′

BMP4 rev: 5′-GTTCTCCAGATGTTCTTCGTGATG-3′

Col2a1 fwd: 5′-AGTGGAAGAGCGGAGACTACTG-3′

Col2a1 rev: 5′-TTGGGGTAGACGCCAAGTCTC-3′

Id1 fwd: 5′-GCGAGATCAGTGCCTTGG-3′

Id1 rev: 5′-CTCCTGAAGGGCTGGAGT-3′

Gli1 fwd: 5′-CAAGTGCACGTTTGAAG-3′

Gli1 rev: 5′-CAACCTTCTTGCTCACACATGTAAG-3′

Dusp6 fwd: 5′-AGTTTTTCCCTGAGGCCATT-3′

Dusp6 rev: 5′-GCATCGTTCATGGACAGGTT-3′

Grem1 fwd: 5′-CCCACGGAAGTGACAGAATGA-3′

Grem1 rev: 5′-AAGCAACGCTCCCACAGTGTA-3′

Jag1 fwd: 5′- GCGGTTGCAGAAGTCAGAGT-3′

Jag1 rev: 5′- AGGCTGTCACCAAGCAACAG -3′

Msx2 fwd: 5′-ATACAGGAGCCCGGCAGATACT-3′

Msx2 rev: 5′-TCCGGTTGGTCTTGTGTTTCC-3′

Spry4 fwd: 5′-TGTGACTCTGCA GCTCCTCAAA-3′

Spry4 rev: 5′-ATGAGGCTGGAGGTCCTGAACT-3′

Sox9 fwd: 5′-CAAGTGTGTGTGCCGTGGATAG-3′

Sox9 rev: 5′-CCAGCCACAGCAGTGAGTAAGAA-3′

Rpl19 fwd: 5′-ACCCTGGCCCGACGG-3′

Rpl19 rev: 5′-TACCCTTTCCTCTTCCCTATGCC-3′

Development • Supplementary information
